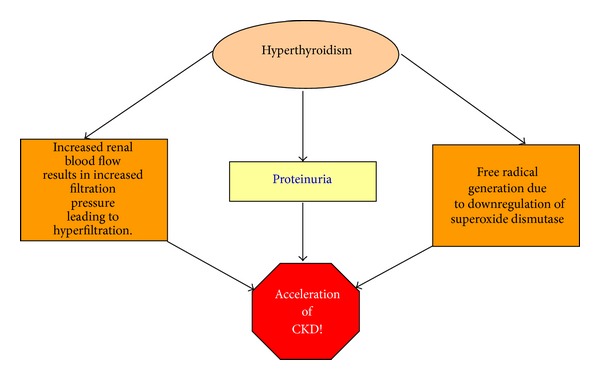# Thyroid Disorders and Chronic Kidney Disease

**DOI:** 10.1155/2014/520281

**Published:** 2014-04-13

**Authors:** Mohamed Mohamedali, Srikanth Reddy Maddika, Anix Vyas, Viswanathan Iyer, Pramil Cheriyath

**Affiliations:** Department of Medicine, Pinnacle Health Affiliated to Penn State Hershey School of Medicine, Harrisburg, PA 17101, USA

## Abstract

Thyroid hormones play a very important role regulating metabolism, development, protein synthesis, and influencing other hormone functions. The two main hormones produced by the thyroid are triiodothyronine (T3) and thyroxine (T4). These hormones can also have significant impact on kidney disease so it is important to consider the physiological association of thyroid dysfunction in relation to chronic kidney disease (CKD). CKD has been known to affect the pituitary-thyroid axis and the peripheral metabolism of thyroid hormones. Low T3 levels are the most common laboratory finding followed by subclinical hypothyroidism in CKD patients. Hyperthyroidism is usually not associated with CKD but has been known to accelerate it. One of the most important links between thyroid disorders and CKD is uremia. Patients who are appropriately treated for thyroid disease have a less chance of developing renal dysfunction. Clinicians need to be very careful in treating patients with low T3 levels who also have an elevation in TSH, as this can lead to a negative nitrogen balance. Thus, clinicians should be well educated on the role of thyroid hormones in relation to CKD so that proper treatment can be delivered to the patient.

## 1. Introduction

The function of the thyroid gland is one of the most important in the human body as it regulates majority of the body's physiological actions. The thyroid produces hormones (T3 and T4) that have many actions including metabolism, development, protein synthesis, and the regulation of many other important hormones. Any dysfunction in the thyroid can affect the production of thyroid hormones (T3 and T4) which can be linked to various pathologies throughout the body. One of the most important conditions that has been less studied is thyroid hormone levels and how they affect the progression of CKD. Disorders in renal function have been seen to coexist with specific levels of thyroid hormone. This study is done to simplify the importance of interactions between thyroid function and kidney disease. This information is essential as it shows a link between two separate conditions. Information obtained from this paper will help to increase clinical knowledge and enable clinicians to provide better management for their patients who have thyroid or kidney dysfunction.

## 2. Methodology

A search for articles was done using PubMed, Google Scholar, Cochran Review, and review of journals within a medical library. Specific searches used were “thyroid function in CKD, hypothyroidism, hyperthyroidism, chronic kidney disease, thyroid hormones in dialysis, nephritic and nephrotic syndrome, thyroid cancer, and glomerulonephritis.” The most relevant and current articles were selected and retrieved as available in their original form or abstracts. All articles were reviewed; relevant data was extracted from the most up to date information. Prior studies and publications were also used.

## 3. Thyroid Disease Epidemiology

Approximately 1 in 13 or 20 million people (7.35%) have thyroid disease in the United States [1]. Thyroid disorders are classified into hypothyroid, hyperthyroid, and a subclinical state. Based on the largest population study* NHANES III*, 4.6% of the US population is suffering from hypothyroidism (0.3% clinical and 4.3% subclinical) and 1.3% from hyperthyroidism (0.5% clinical and 0.7% subclinical) [2]. Both hypothyroidism and hyperthyroidism account for a high level of morbidity in the United States.

## 4. Chronic Kidney Disease

CKD is usually a progressive, irreversible condition that is the 8th leading cause of death in the United States [3]. According to the population study, 1 in 10 American adults (more than 30 million people) suffer from some level of CKD [3]. Risk factors for CKD include diabetes, hypertension, hyperlipidemia, and thyroid disorders.

## 5. Thyroid Physiology (Figure 1)

T3 and T4 play a critical role in cell differentiation during development and help maintain metabolic homeostasis in the adults [4].

## 6. Thyrotropin Releasing Hormone (TRH)

TRH is a very small tripeptide amide L-pyroglutamyl-L-histidyl-L-prolineamide (L-PHP) [5]. TRH release is influenced by thyroid hormones in circulation and control from the hypothalamus. Once TRH is released from the hypothalamus, it travels to the anterior pituitary, where it interacts with TRH receptors (C-phosphoinositide pathway) to cause the release of thyroid stimulating hormone (TSH) (3).

## 7. Thyroid Stimulating Hormone (TSH)

TSH is a 28 to 30 kDa glycoprotein subset of the cystine-knot growth factor super family [6]. It is produced from the basophilic cells of the anterior pituitary gland. After being released from the anterior pituitary, TSH travels to the thyroid gland and binds with TSH receptors on thyroid cells. This physiological action activates the second messenger pathway resulting in thyroid gene expression and the release of T3/T4.

## 8. Thyrotropin Molecular Structure (Figure 2)

The structure of the thyrotropin hormone consists of a human *α* subunit and *β* subunit located on chromosomes 1 and 6, respectively. The alpha subunit gene contains 4 exons and 3 introns which is twice larger than the *β* subunit gene containing 3 exons and 2 introns [6]. The alpha subunit serves as the effector region for stimulation of the second messenger pathways. The physiological action of the *β* subunit plays an important role in determining thyrotropin's receptor specificity.

## 9. CKD in Relation to Thyroid Disorders (Figure 3)

As mentioned, CKD affects the hypothalamus-pituitary-thyroid axis and the peripheral metabolism of thyroid hormone. Low T3 is the most common laboratory finding and subclinical hypothyroidism is most common thyroid disorder found in CKD patients [7]. TSH levels are usually normal with an altered circadian rhythm (comprised of TSH bioactivity). In uremia, the pituitary receptor response to TRH is blunted causing a decrease in TSH release. The response of TSH to TRH is delayed because of the decreased clearance and the increase of half-life of TSH [7]. Abnormal serum constituents found in uremic conditions can also displace T3 and T4 from normal protein binding sites. Normal or low levels of T4 may be due to the monodeiodinase action occurring in the inner benzene ring instead of outer ring of T4, resulting in the formation of reverse T3. Reverse T3 levels, however, are found to be normal in CKD patients because it moves from the vascular space to extra vascular and intracellular spaces [7]. Transient increases in T4 levels are usually seen after hemodialysis. This effect is mainly due to the use of heparin as an anticoagulant which inhibits T4 binding to proteins and leads to an increase in T4 levels [7].

Low T3 levels in CKD may be due to the iodothyronine deiodinase (helps in T3 synthesis from T4) affected by fasting, chronic metabolic acidosis, and chronic protein malnutrition seen in CKD. Such factors influence the proteins binding to T3 [7]. Low T3 levels in CKD may also be due to the decreased peripheral (extra thyroidal) conversion from T4 to T3 due to decreased clearance of the inflammatory cytokines such as TNF-alpha and IL-1. These cytokines inhibit expression of 1 5′-deiodinase that helped convert T4 to T3 [8]. Low free T3 levels have shown to be an independent predictor of mortality in hemodialysis patients [9]. Low T3 levels prior to renal transplant are associated with posttransplant risks of graft loss [7]. All clinicians are advised to check T3 levels before renal transplantation. Low T3 levels in CKD may not be able to increase TSH levels. Experimental evidence suggests that, in uremia, the sensitivity of thyrotrophs is increased. This may account for the resetting of the central thyrostat indicating a lower level of the circulating thyroid hormones and, in turn, affect the negative feedback inhibition [10]. In CKD, physiological compensation for low T3/T4 (with normal TSH levels) causes a reduction in protein catabolism which increases the nitrogen waste overload.

## 10. Goiter in CKD

There is an increased prevalence of goiter (0–9%) in patients with CKD. This may be due to the decreased clearance of the inorganic iodides, causing a hypertrophic effect on the thyroid gland tissue leading to goiter [7]. A decreased clearance of goitrogenic substances like aryl acid due to CKD may also be a factor [8]. Research has shown that increased serum iodine levels can result in prolongation of the Wolff-Chaikoff effect.

## 11. Subclinical Hypothyroidism

Subclinical hypothyroidism is defined as an elevation in serum TSH concentration (normal range 5–10 *μ*IU/mL) in conjunction with a normal serum free T4 concentration. With the decline in GFR, the prevalence of subclinical hypothyroidism increases consistently. One study showed that approximately 18% of the patients with CKD not requiring dialysis have subclinical primary hypothyroidism. This finding is independently associated with a progressively lower estimated GFR. The prevalence of subclinical primary hypothyroidism increased from 7% to 17.9% in individuals whose GFR has decreased from ≥90 mL/min to 60 mL/min [11]. Some researchers reported that hypothyroidism can be corrected with restriction of dietary iodine in uremic patients on dialysis which decreases the need for hormone replacement therapy. In one clinical trial, the overall rate of a decline in the estimated GFR was significantly greater in those not treated with thyroid hormones compared to those who were treated with thyroid hormones.

## 12. Hyperthyroidism (Figure 4)

The prevalence of hyperthyroidism in CKD patients is the same as it is with the general population; thus CKD is not directly associated with hyperthyroidism. However, it is important to understand that aspects of hyperthyroidism can indeed accelerate CKD. These mechanisms are the following:

- increased renal blood flow seen in hyperthyroidism results in intraglomerular hypertension, leading to increased filtration pressure and consequent hyperfiltration. Proteinuria seen in hyperthyroidism is known to cause direct renal injury;
- increased mitochondrial energy metabolism along with downregulation of superoxide dismutase, which occurs in hyperthyroidism, contributes to an increased free radical generation that causes renal injury;
- oxidative stress also contributes to hypertension in hyperthyroidism, which contributes to CKD progression [7].

## 13. Thyroid Disorders in Glomerular Diseases

Thyroid diseases including both hypo- and hyperthyroidism are associated with several types of glomerulonephritis. The types of glomerulonephritis seen in thyroid disease are membranous, IgA, mesangiocapillary, membranoproliferative, and minimal change glomerulonephritis. Among these, the most frequent is membranous glomerulonephritis [12]. The two main histological changes seen are a thickened glomerular basement membrane (GBM) due to immune complex deposition and an increased mesangial and endocapillary cellularity [13]. The pathophysiology links between thyroid dysfunction and glomerulonephritis involve proteinuria and formation of immune complexes [12]. This association is extremely common in autoimmune thyroiditis. Approximately up to 50% of patients with autoimmune thyroiditis have the presence of immune complexes. These complexes are mainly responsible for the alteration of the renal function by depositing on the basement membrane of the glomeruli. Some studies have also reported a deposition of thyroglobulin in the basement membrane of the glomeruli. In addition to thyroid diseases, similar effects are also seen in other autoimmune disorders such as systemic lupus erythematosus (SLE) and diabetes [12].

## 14. Nephrotic Syndrome

Changes in the serum levels of thyroid hormone can affect nephrotic syndrome in many ways. Due to proteinuria, there is a loss of many binding proteins including thyroxine-binding globulin (TBG), transthyretin or prealbumin, and albumin [12]. Due to losses of these proteins, there is a reduction in serum T4 and total T3 levels. In most circumstances, patients are euthyroid because the thyroid is able to compensate for the proteinuria and free T3 and T4 levels are normal [14]. The role of using levothyroxine to replace of thyroid hormones remains controversial.

## 15. Thyroid Cancer and Kidney Disease

The incidence of thyroid cancer has increased worldwide. Women are 3 times more likely to be diagnosed with thyroid cancer [12]. Patients with thyroid cancer are also predisposed to other types of cancer including renal cell carcinoma. Many studies are looking at the relationship between thyroid hormones and reproductive cancers. Other types include parenchymal epithelial tumors, oncocytoma, collecting duct tumors, and renal sarcoma. Kidney metastasis from thyroid carcinomas is seen in papillary [15–21], follicular [22–26], and anaplastic thyroid cancers [27]. Kidney tumors can also metastasize to the thyroid gland.

## 16. Effects of Dialysis on Thyroid Hormones Hemodialysis

Most patients on hemodialysis (HD) are euthyroid [12]. Systemic acidosis, time on dialysis, markers of endothelial damage, and inflammation from HD are associated with low T3 levels [12]. Low total T4 levels with increased free T4 levels are seen as heparin inhibits T4 binding to proteins, thereby increasing a free T4 fraction in these patients. TSH is elevated in 20% of patients on HD usually in the range of 5–20 mU/L [7]. HD affects the cellular transport of TSH which might act as a compensatory mechanism for maintaining an euthyroid status [12].

## 17. Peritoneal Dialysis

Peritoneal dialysis (PD) is usually associated with low T3 levels and subclinical hypothyroidism [8]. Low T3 levels might be due to inflammation and malnutrition in PD, as there is an association between free T3, CRP, and serum albumin [28]. Subclinical hypothyroidism may be due to a decrease in iodide clearance. Iodide clearance is done mainly by glomerular filtration; thus, in advanced CKD, iodide excretion is diminished with subsequent elevation in plasma inorganic iodide concentration. Such increases in total body inorganic iodide may block thyroid hormone production which may explain the high incidence of subclinical hypothyroidism in CKD patients. The T4 and T3 losses are minor (10% and 1%, resp.) and easily compensated in PD [7]. Thyroid hormone supplementation is not necessary in patients on PD.

Thyroxine-binding globulin (TBG) is lost along with T4 and T3 in the PD; however, TBG levels are normal. Recent studies have demonstrated that subclinical hypothyroidism is associated with an increased risk for cardiovascular disease and can cause mortality in CKD patients.

## 18. Renal Transplant

Renal transplantation is known to normalize thyroid hormone levels. Gradually within 3 to 4 months, low T3 and T4 return to normal in renal transplant patients. During the first few months of transplant, T4 is reduced less than the pretransplant stage and gradually returns to normal [8]. Normally, free T3 and thyroid volume will correlate with graft function in posttransplantation patients. The low T3 levels before transplant are associated with future risk of graft loss, and thyroid hormone supplementation will not be useful; treating these patients with thyroid hormones is not necessary [8].

## 19. Conclusion

Thyroid disorders and CKD are independently some of the most prominent medical conditions found in patients in the United States. Due to the high prevalence of both, it is important to consider the physiological association of thyroid dysfunction in relation to kidney disease. The most common changes in CKD relating to the thyroid gland are of low T3 levels and subclinical hypothyroidism. The prevalence of subclinical hypothyroidism increases consistently in patients who have a decline in GFR. Low T3, normal to reduced T4 levels, and normal TSH often result in increased thyroid gland volume. In turn, a decrease in renal function also accounts for an ineffective clearance of abnormal serum constituents, inflammatory cytokines, iodide excretion, and an increase of nitrogen conservation. All of these factors have been clinically proven to affect the normal physiology and metabolism of thyroid hormones. Hyperthyroidism is usually not associated with CKD but is known to accelerate it. It is very important to consider all clinical features and thyroid manifestations in those patients with CKD. As seen in many evidence-based studies and current clinical cases, there are distinct relationships in thyroid dysfunction and kidney disease and vice versa.

Clinicians, including nephrologists, must consider the dangers of thyroid disease and its appropriate treatment in conjunction to treating CKD. Patients who receive appropriate treatment for their thyroid disease have a decreased chance of developing or exacerbating renal dysfunction. However, treating patients with a mild elevation of TSH (less than 20 IU/mL) results in a negative nitrogen balance by increased muscle catabolism. Clinicians should look for low T3 levels in patients prior to renal transplant as low levels are associated with renal graft loss.

## Figures and Tables

**Figure 1:**
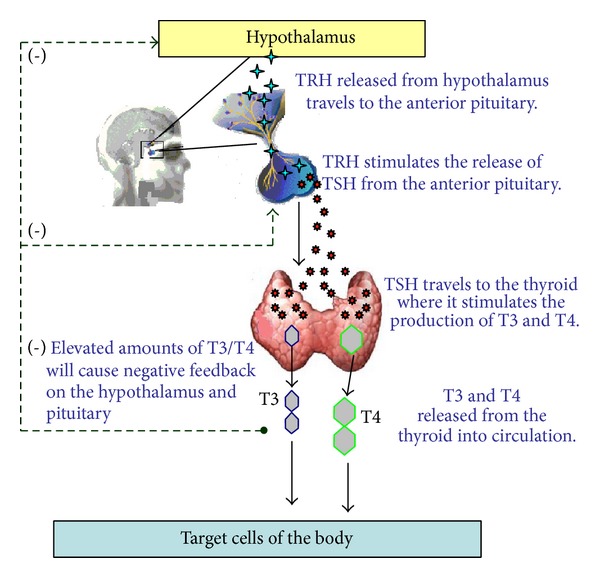


**Figure 2:**
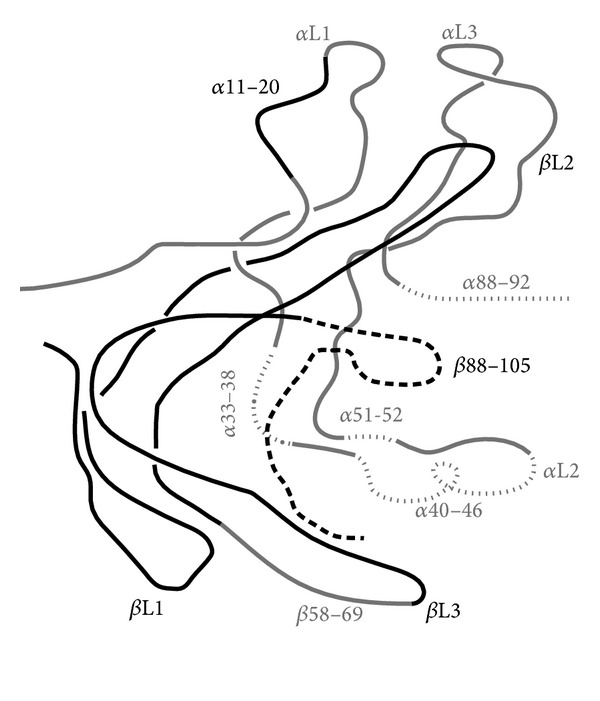
Structure of thyrotropin.

**Figure 3:**
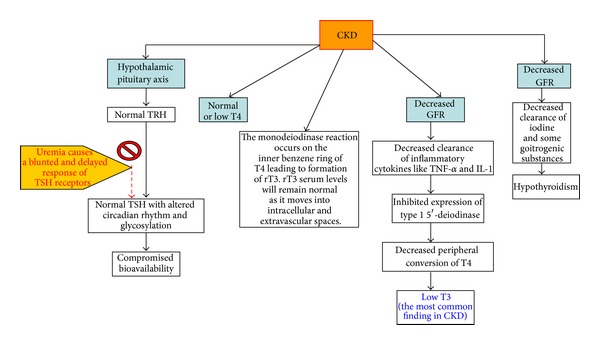


**Figure 4:**